# Sarcopenia in idiopathic pulmonary fibrosis: a prospective study exploring prevalence, associated factors and diagnostic approach

**DOI:** 10.1186/s12931-022-02159-7

**Published:** 2022-09-03

**Authors:** Paola Faverio, Alessia Fumagalli, Sara Conti, Fabiana Madotto, Francesco Bini, Sergio Harari, Michele Mondoni, Tiberio Oggionni, Emanuela Barisione, Paolo Ceruti, Maria Chiara Papetti, Bruno Dino Bodini, Antonella Caminati, Angela Valentino, Stefano Centanni, Paola Lanzi, Matteo Della Zoppa, Silvia Crotti, Marco Grosso, Samir Giuseppe Sukkar, Denise Modina, Marco Andreoli, Roberta Nicali, Giulia Suigo, Sara Busnelli, Giuseppe Paciocco, Sara Lettieri, Lorenzo Giovanni Mantovani, Giancarlo Cesana, Alberto Pesci, Fabrizio Luppi

**Affiliations:** 1 School of Medicine and Surgery, University of Milano Bicocca, Respiratory Unit, San Gerardo Hospital, ASST Monza, Monza, Italy; 2 Pulmonary Rehabilitation Unit, Research Hospital of Casatenovo, Italian National Research Centre on Aging (INRCA), Casatenovo, LC Italy; 3 Research Center on Public Health, Department of Medicine and Surgery, Università degli Studi di Milano-Bicocca, Monza, Italy; 4 Dipartimento di Anestesia, Rianimazione ed Emergenza Urgenza, Fondazione IRCCS Ca’ Granda Ospedale Maggiore Policlinico, Milano, Italy; 5 UOC Pneumologia, Ospedale G. Salvini, ASST Rhodense, Garbagnate Milanese, MI Italy; 6 UOC di Pneumologia e Terapia Semi-Intensiva Respiratoria - Servizio di Fisiopatologia Respiratoria ed Emodinamica Polmonare, Ospedale San Giuseppe, MultiMedica IRCCS, Milano, Italy; 7 Department of Clinical Sciences and Community Health, University of Milan, Milano, Italy; 8 Department of Health Diseases, Università degli Studi di Milano Respiratory Unit, ASST Santi Paolo e Carlo, Milan, Italy; 9 Pulmonology Unit, Fondazione IRCCS Policlinico San Matteo, Pavia, Italy; 10 UOC Pneumologia Interventistica, IRCCS Policlinico San Martino, Genoa, Italy; 11 UO Pneumologia, ASST Spedali Civili di Brescia, Brescia, Italy; 12 UOC Di Diabetologia e Malattie Metaboliche, IRCCS Multimedica, Milano, Italy; 13 SSD Servizio Dietetico e Nutrizione Clinica, ASST Santi Paolo e Carlo, Milano, Italy; 14 Clinical Nutrition and Dietetics Unit, Fondazione IRCCS Policlinico San Matteo, Pavia, Italy; 15 UOD Dietetica e Nutrizione Clinica, IRCCS Ospedale Policlinico San Martino, Genova, Italy; 16 USD Servizio Dietetica e Nutrizione Clinica, ASST di Brescia, Brescia, Italy; 17 UOC Pneumologia, Azienda Ospedaliero Universitaria Maggiore della Carità, Novara, Italy; 18 UO Pneumologia, Azienda Ospedaliera di Circolo, Busto Arsizio, VA Italy

**Keywords:** Idiopathic pulmonary fibrosis, Sarcopenia, Hand grip, Bioimpedance analysis, Gait speed

## Abstract

**Background:**

Sarcopenia gained importance in the evaluation of patients with chronic respiratory diseases, including idiopathic pulmonary fibrosis (IPF), since it may impact negatively on clinical outcomes.

**Aim:**

Aim of this study is to evaluate the prevalence and factors associated with sarcopenia, defined according to the European Working Group on Sarcopenia in Older People 2 (EWGSOP2) 2019 definition, and to evaluate the prevalence of the single criteria that define the EWGSOP2 definition (muscle strength, muscle quantity and physical performance), in a cohort of consecutive patients with IPF prospectively followed up in 9 hospitals in Northern Italy between December 2018 and May 2021.

**Methods:**

Enrolled patients underwent an extensive pulmonary and nutritional assessment, including bioelectrical impedance analysis, dynamometry and 4-m gait speed test, both at IPF diagnosis and at 6-month follow-up.

**Results:**

Out of the 83 patients (81% males, mean age 72.5 years) with IPF at disease diagnosis enrolled in the study, 19 (22.9%) showed sarcopenia, including 2 (2.4%) with severe sarcopenia, 5 (6.0%) with confirmed sarcopenia and 12 (14.5%) with probable sarcopenia. Sarcopenia was associated with a significantly higher severity of the disease and sedentary lifestyle, while no differences were observed in regards to body mass index, history of weight loss and comorbidities between patients with and without sarcopenia. Out of the 64 patients without sarcopenia at baseline, 16 cases showed alteration of muscle quantity and/or physical performance. In the 51 patients with complete data at 6-month follow-up, there were no cases of severe sarcopenia, 1 case (2.0%) showed confirmed sarcopenia, while the prevalence of probable sarcopenia was 19.6% (10 cases). No differences in regards to antifibrotic treatment received and onset of gastrointestinal side effects were observed between patients with and without sarcopenia at follow-up.

**Conclusions:**

The prevalence of sarcopenia in patients with IPF both at diagnosis and at 6-month follow-up was low but not negligible and was associated with higher severity of the disease and sedentary lifestyle. In IPF patients, a comprehensive diagnostic work-up including all the criteria defining the EWGSOP2 definition might be more useful than a series testing for prompt recognition of nutritional and physical performance abnormalities.

Sarcopenia has recently gained importance in the evaluation of patients with chronic respiratory diseases [1], although its evaluation is hampered by the absence of standardised definition criteria [2]. In idiopathic pulmonary fibrosis (IPF) low muscle mass, evaluated through bioimpedance analysis (BIA), has been associated with a worse prognosis [3–5]. Furthermore, patients with overt IPF may experience a downward spiral: in fact the restrictive pattern and hypoxia secondary to the fibrotic alterations may lead to increased respiratory muscle load and dyspnea that worsen the sedentary lifestyle leading to muscle deconditioning and, ultimately, sarcopenia [6].

A new definition published in 2019 by the European Working Group on Sarcopenia in Older People 2 (EWGSOP2)*,* focusing on muscle weakness as principal determinant more than muscle mass, differentiate sarcopenia in 3 stages: (1) probable sarcopenia if only low muscle strength, dynapenia, is present (hand grip < 27 kg for men and < 16 kg for women); (2) confirmed sarcopenia if low muscle quantity or quality is also present (appendicular skeletal muscle mass (ASMM) < 20 kg for men and ASMM < 15 kg for women); (3) severe sarcopenia if both prior criteria and low physical performance, defined as gait speed ≤ 0.8 m/sec, occur [7]. The previous definition has been developed and applied as a series testing, in which the three measurements are performed sequentially only in case of positivity of the prior one.

In a recent multicenter observational study, we highlighted how early signs of nutritional and physical performance impairment, including reduction of gait speed and hand grip strength, can already be observed in patients with IPF at the time of the diagnosis [8]. However, the clinical impact as well as the factors associated with these early alterations and overt sarcopenia are not completely understood.

Aim of this study is to evaluate the prevalence and factors associated with sarcopenia, defined according to EWGSOP2 2019 definition, and to evaluate the prevalence of the single criteria that define the EWGSOP2 score, in a cohort of consecutive patients with IPF prospectively followed up in 9 hospitals in Northern Italy between December 2018 and May 2021.

Eighty-three consecutive patients (81% males, mean age 72.5 years) at the time of diagnosis with complete clinical data were included in the study and performed extensive respiratory and nutritional assessments both at diagnosis (before antifibrotic initiation) and after 6 months. Respiratory assessment included complete pulmonary function tests (PFTs), diffusing capacity for carbon monoxide (DLCO), six-minute walking test (6MWT) and Gender-Age-Physiology (GAP) score; nutritional assessment included anthropometric measurements, International Physical Activity Questionnaire (IPAQ)), BIA, dynamometry and 4-m gait speed test. ASMM was calculated according to the formula reported by Sergi et al*.* [9] and patients were classified based on EWGSOP2 2019 definition. Patients were stratified into two groups: those at any stage of sarcopenia and those without sarcopenia at baseline. All the baseline characteristics of these two groups were synthesized through absolute and relative frequencies for categorical variables and mean and standard deviation (SD) for continuous variables. The two groups were then compared using chi-square test and Fisher’s exact test for categorical characteristics and Wilcoxon rank-sum test for continuous ones. For all tests, the significance level was 0.05. Alluvial plots, which describe the variation of one or more characteristics in time in form of a flow, were used to compare sarcopenia or the presence of each criteria for its definition at baseline and after 6 months. Analyses were performed with statistical software SAS version 9.4 (SAS Institute, Cary, NC, USA) and R version 4.0.5 (R Project for Statistical Computing, www.R-project.org). This study received Ethics Committee approval (#1867, ASST Monza, October 2018) and was registered on clinicaltrial.gov (NCT03770845—NutrIPF study).

At disease diagnosis, 2 (2.4%) patients fulfilled the criteria for severe sarcopenia, 5 (6.0%) patients for confirmed sarcopenia and 12 (14.5%) patients had probable sarcopenia.

Compared to subjects without sarcopenia (64, 77.1%), patients with sarcopenia of any degree (19, 22.9%) at baseline were significantly older, with higher GAP stage, lower mean percentage values of Forced Vital Capacity (FVC) and DLCO and a lower level of daily physical activity according to the IPAQ questionnaire: 12 (63.2%) cases in the sarcopenia group *vs* 26 (41.0%) non-sarcopenic cases had an inactive lifestyle (≤ 700 MET), Table 1. No differences were observed between patients with and without sarcopenia in regard to gender, body mass index (BMI), comorbidities, distance walked at 6MWT, and history of weight loss > 5% in the prior 6 months, Table 1.

**Table 1:** Comparison in demographic and clinical characteristics between sarcopenic and non-sarcopenic subjects at IPF diagnosis

	Sarcopenia at IPF diagnosis		Total (N = 83)	p-value
	No (N = 64)	Yes (N = 19)		
Demographics and clinical characteristics at IPF diagnosis				
Male gender—N(%)	50 (78.13%)	17 (89.47%)	67 (80.72%)	0.3404
Age at enrollment—Mean (SD)	71.6 (6.9)	75.6 (6.1)	72.5 (6.9)	0.0162
BMI—Mean (SD)	28.0 (3.9)	26.6 (4.4)	27.6 (4.0)	0.1198
GAP stage*—N(%)				0.0223
1	22 (34.38%)	1 (5.26%)	23 (27.71%)	
2	29 (45.31%)	15 (78.95%)	44 (53.01%)	
3	12 (18.75%)	3 (15.79%)	15 (18.07%)	
FVC%—Mean (SD)	89.5 (22.1)	73.2 (12.6)	85.7 (21.3)	0.0015
DLCO%—Mean (SD)	56.5 (19.0)	46.8 (15.4)	54.2 (18.6)	0.0442
6MWT (meters walked)*—Mean (SD)	409.4 (114.0)	382.6 (102.0)	403.2 (111.3)	0.3387
Comorbidities—N(%)				
Arrhythmia	6 (9.38%)	2 (10.53%)	8 (9.64%)	1
Congestive heart failure	2 (3.13%)	0 (0.00%)	2 (2.41%)	1
Coronary artery disease	10 (15.63%)	4 (21.05%)	14 (16.87%)	0.7276
Previous solid neoplasm	10 (15.63%)	3 (15.79%)	13 (15.66%)	1
Dysthyroidism				0.7374
Hyperthyroidism	2 (3.13%)	0 (0.00%)	2 (2.41%)	
Hypothyroidism	2 (3.13%)	1 (5.26%)	3 (3.61%)	
MRGE	17 (26.56%)	2 (10.53%)	19 (22.89%)	0.2158
Osteoporosis	5 (7.81%)	1 (5.26%)	6 (7.23%)	1
Sleep apnea	2 (3.13%)	2 (10.53%)	4 (4.82%)	0.2235
Anxiety an depression	5 (7.81%)	1 (5.26%)	6 (7.23%)	1
Chronic liver disease	5 (7.81%)	1 (5.26%)	6 (7.23%)	1
Pulmonary hypertension	0 (0.00%)	1 (5.26%)	1 (1.21%)	0.2289
Vasculopathy	8 (12.50%)	1 (5.26%)	9 (10.84%)	0.6768
Diabetes	7 (10.94%)	3 (15.79%)	10 (12.05%)	0.6888
Hypertension	26 (40.63%)	7 (36.84%)	33 (39.76%)	1
N comorbidities				0.7673
0–2	38 (59.38%)	12 (63.16%)	50 (60.24%)	
≥ 3	26 (40.63%)	7 (36.84%)	33 (39.76%)	
Physical performance and nutritional risk screening				
Physical activity, IPAQ				0.0028
Mean MET (SD)	2.382 (3.296)	9.92 (1.734)	2.077 (3.069)	
% weight loss in 6 months > 5%—N(%)	6 (9.38%)	3 (15.79%)	9 (10.84%)	0.421

*BMI* body mass index, *GAP* Gender-Age-Physiology, *FVC* Forced Vital Capacity, *DLCO* diffusing capacity for carbon monoxide, *6MWT* six-minute walking test, *IPAQ* International Physical Activity Questionnaire

Sixty-five out of 83 patients completed the follow-up at a median (IQR) 6.4 (5.6–7.5) months from disease diagnosis, while 15 patients were lost and 3 died during follow-up, due to worsening of IPF. No differences in regard to age, gender, GAP stage, PFTs and IPAQ at baseline were observed between patients who completed and those lost to follow-up, Table 2. Among those who died, one was non-sarcopenic, one had probable sarcopenia and one had confirmed sarcopenia. In the 51 patients with complete data in whom the evaluation of sarcopenia could be performed, there were no cases of severe sarcopenia, 1 case (2.0%) showed confirmed sarcopenia, while the prevalence of probable sarcopenia was 19.6% (10 cases). No differences in regards to antifibrotic treatment received, dose reduction of specific therapy and onset of gastrointestinal side effects were observed between patients with and without sarcopenia at follow-up. In particular, among the patients with sarcopenia 1 received pirfenidone and 9 nintedanib, while among patients without sarcopenia 12 received pirfenidone, 19 nintedanib and 1 switched from pirfenidone to nintedanib. Comparison in the sarcopenic status between baseline and 6-month follow-up is summarised in Fig. 1a. In addition, patients with sarcopenia at 6-month follow-up were significantly older and with a more sedentary lifestyle compared to those without sarcopenia, Table 2.

**Table 2:** Comparison between lost to follow-up and others and between sarcopenic and non-sarcopenic patients at 6-month follow-up

	Lost to follow-up at 6 months		Total (N = 83)	p-value
	No (N = 68)	Yes (N = 15)		
Age at enrolment, Mean (SD)	72.0 (7.0)	75.1 (6.0)	72.5 (6.9)	0.0879
GAP stage*				0.1206
1	21 (30.88%)	2 (13.33%)	23 (27.71%)	
2	36 (52.94%)	8 (53.33%)	44 (53, 01%)	
3	11 (16.18%)	4 (26.67%)	15 (18, 07%)	
FVC%*, Mean (SD)	86.8 (21.9)	80.5 (18.1)	85.7 (21.3)	0.4447
DLCO%, Mean (SD)	53.5 (18.4)	57.5 (20.1)	54.2 (18.6)	0.3818
IPAQ*, Mean MET (SD)	2.2330 (3.1908)	1.3804 (2.4177)	2.0771 (3.0685)	0.0788

	Sarcopenia at 6-month follow-up		Total (N = 51)	p-value
	No (N = 40)	Yes (N = 11)		
Age at enrolment, Mean (SD)	69.5 (7.2)	77.0 (5.6)	71.1 (7.5)	0.0021
Pneumological visit at 6-month follow-up				
GAP stage				0.8476
1	16 (40.00%)	3 (27.27%)	19 (37.25%)	
2	14 (35.00%)	5 (45.45%)	19 (37.25%)	
3	5 (12.50%)	2 (18.18%)	7 (13.73%)	
Missing	5 (12.50%)	1 (9.09%)	6 (11.76%)	
FVC%°, Mean (SD)	90.9 (27.0)	88.2 (15.0)	90.3 (24.6)	1
DLCO%**, Mean (SD)	52.6 (17.1)	45.4 (15.5)	51.0 (16.9)	0.2866
IPAQ°°, Mean MET (SD)	2.449.2 (3.4330)	299.1 (248.5)	2.0424 (3.1989)	0.0079

*1 missing value, °3 missing values, **4 missing values, °°10 missing values. *GAP* Gender-Age-Physiology, *FVC* Forced Vital Capacity; *DLCO* diffusing capacity for carbon monoxide, *IPAQ* International Physical Activity Questionnaire

**Fig. 1.**
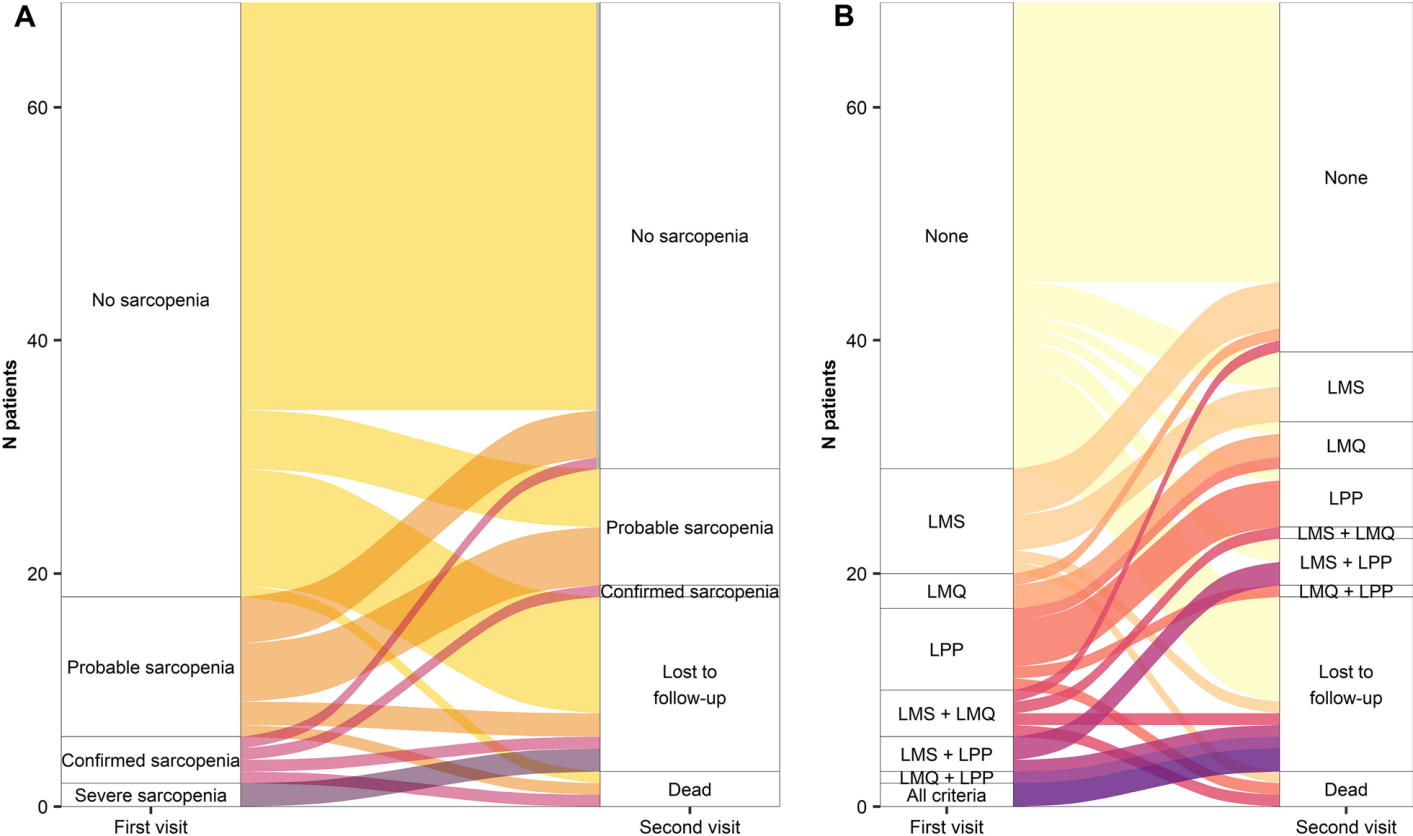
**a** Alluvial plot of sarcopenia during the first and the second nutritional visit; **b** alluvial plot of the combination of criteria for sarcopenia during the first and the second nutritional visit. *LMS* Low muscle strength, *LMQ* Low muscle quantity, *LPP* Low physical performance

When applying the criteria used for the definition of sarcopenia at baseline as a parallel testing, 4 (4.8%), 10 (12.0%) and 2 (2.4%) patients without low muscle strength had low muscle quantity, low physical performance and the contemporary presence of both low muscle quantity and low physical performance, respectively, Fig. 1b. These changes would have been missed applying the criteria as a series testing. Furthermore, when looking at the criteria indicated in the EWGSOP2 definition of sarcopenia, we observed a high variability between baseline and 6-month visit in regards to single criteria, e.g. low muscle strength, and associations of criteria, Fig. 1b. These findings may suggest that in frail patients at high risk of progressive pulmonary deterioration a diagnostic approach with high positive predictive value but low sensitivity, as series testing, might hamper the ability to make an early diagnosis of nutritional and physical performance impairment and to start an early treatment.

Different speculations can also be raised on the high variability observed among the visits. First of all, the EWGSOP2 2019 definition may have a poor repeatability in a population of IPF patients; secondly, the definition may not be specific enough for this cohort of patients. On the other hand, also the single criteria composing the definition may vary among time points and this may be due to (1) a successful therapeutic or life-style approach between visits that may revert the nutritional and/or physical performance impairment; (2) the cut-off points used for the main variables (e.g. hand-grip strength) which may lead to a change in the sarcopenia category for a small change in the scores of the single variables. These are also some of the reasons why we suggest to perfom a complete nutritional evaluation, assessing in parallel both muscle strength, quantity and physical performance, instead of a series testing, to better phenotype the patient in regard to nutritional and physical performance status, as reported both for IPF and chronic obstructive pulmonary disease patients [8, 10].

Patients with sarcopenia at baseline were also those with higher severity of the disease, evaluated through GAP index, and more sedentary lifestyle. Although IPF is indisputably considered a progressive disease, in contrast sarcopenia may be approached with nutritional and rehabilitation programs.

Despite the growing evidence on the positive impact of pulmonary rehabilitation in patients with IPF [11], only 2 patients in our cohort underwent to such intervention.

Recent literature has evaluated the prevalence of dynapenia and sarcopenia in patients with fibrosing interstitial lung diseases at various stages [12–14].

A recent study by Hanada et al*.* applied the diagnostic criteria of the Asian Working Group for Sarcopenia 2019 in a cohort of 78 patients with interstitial lung diseases (ILD) of Asian ethnicity and observed a prevalence of sarcopenia of 32%, higher than the one we observed in our cohort of patients with IPF at diagnosis [12]; however, these differences may be due to the different ethnicity of the study population (in our study all patients were Caucasian) and to the different underlying pulmonary disease. On the contrary, Bocchino et al*.* in a cohort of Italian patients with IPF at various stages of the disease reported a prevalence of dynapenia defined in accordance to the EWGSOP2 criteria (hand grip < 27 kg for men and < 16 kg for women) exactly comparable to ours (22.9 and 21.6% in our cohort at disease diagnosis and at 6-month follow-up, respectively, and 23.5% in the cohort by Bocchino et al*.*) [13]*.* Furthermore, similarly to our study, Bocchino et al*.* observed an association between disease severity and dynapenia, and Guler et al*.* observed that ILD severity impacted on both grip strength, gait speed and body composition [13, 14].

Among the main strengths of our study we acknowledge the prospective multicentric design, which included specialist IPF clinics in both university and non-university hospitals, that increased the generalizability of the results.

Our study presents also some limitations: the main one is the number of patients lost lo follow-up; however, part of the study was performed during the COVID-19 pandemic with major limitations in following-up IPF patients and this may have contributed to the lost to follow-up and the limited access to pulmonary rehabilitation centers.

In conclusion, the prevalence of sarcopenia in patients with IPF at diagnosis is low but not negligible (23% of cases) and it is associated with a significantly higher severity of the disease and sedentary lifestyle. In IPF patients, a comprehensive diagnostic work-up including both muscle strength, muscle quantity and physical performance may prove to be more useful than a series testing for prompt recognition and early nutritional impairment.

## Data Availability

Individual participant data referring to this article (i.e. text, tables and figures) will be made available upon reasonable request. The study protocol will be made available for researchers who provide a methodologically sound proposal. Proposals should be directed to paola.faverio@unimib.it.

## References

[1] Bone AE, Hepgul N, Kon S (2017). Sarcopenia and frailty in chronic respiratory disease. Chron Respir Dis.

[2] Sepúlveda-Loyola W, Osadnik C, Phu S (2020). Diagnosis, prevalence, and clinical impact of sarcopenia in COPD: a systematic review and meta-analysis. J Cachexia Sarcopenia Muscle.

[3] Suzuki Y, Aono Y, Kono M (2021). Cause of mortality and sarcopenia in patients with idiopathic pulmonary fibrosis receiving antifibrotic therapy. Respirology.

[4] Nakano A, Ohkubo H, Taniguchi H (2020). Early decrease in erector spinae muscle area and future risk of mortality in idiopathic pulmonary fibrosis. Sci Rep.

[5] Moon SW, Choi JS, Lee SH (2019). Thoracic skeletal muscle quantification: low muscle mass is related with worse prognosis in idiopathic pulmonary fibrosis patients. Respir Res.

[6] Faverio P, Bocchino M, Caminati A (2020). Nutrition in patients with idiopathic pulmonary fibrosis: critical issues analysis and future research directions. Nutrients.

[7] Cruz-Jentoft AJ, Bahat G, Bauer J (2019). Sarcopenia: revised European consensus on definition and diagnosis. Age Ageing.

[8] Faverio P, Fumagalli A, Conti S (2022). Nutritional assessment in idiopathic pulmonary fibrosis: a prospective multicentre study. ERJ Open Res.

[9] Sergi G, De Rui M, Veronese N (2015). Assessing appendicular skeletal muscle mass with bioelectrical impedance analysis in free-living Caucasian older adults. Clin Nutr.

[10] Schols AM, Ferreira IM, Franssen FM (2014). Nutritional assessment and therapy in COPD: a European Respiratory Society statement. Eur Respir J.

[11] Yu X, Li X, Wang L (2019). Pulmonary rehabilitation for exercise tolerance and quality of life in IPF patients: a systematic review and meta-analysis. Biomed Res Int.

[12] Hanada M, Sakamoto N, Ishimoto H (2022). A comparative study of the sarcopenia screening in older patients with interstitial lung disease. BMC Pulm Med.

[13] Bocchino M, Alicante P, Capitelli L (2021). Dynapenia is highly prevalent in older patients with advanced idiopathic pulmonary fibrosis. Sci Rep.

[14] Guler SA, Hur SA, Lear SA (2019). Body composition, muscle function, and physical performance in fibrotic interstitial lung disease: a prospective cohort study. Respir Res.

